# Pharmacokinetics of injectable favipiravir: phase I, randomized, double-blind, placebo-controlled study in healthy East Asian subjects

**DOI:** 10.1128/aac.00463-26

**Published:** 2026-07-21

**Authors:** Hiroshige Mikamo, Masahiro Nakabayashi, Tsutomu Sakurai

**Affiliations:** 1 Department of Clinical Infectious Diseases, Asahi University Hospital 73507, Gifu, Japan; 2 Department of Clinical Infectious Diseases, Aichi Medical University 12703, Nagakute, Aichi, Japan; 3 Development Division, FUJIFILM Toyama Chemical Co., Ltd 13417 , Chuo City, Tokyo, Japan; Chinese Academy of Medical Sciences & Peking Union Medical College, Beijing, China

**Keywords:** favipiravir, injectable formulation, phase I, pharmacokinetics, mechanism-based inhibition, safety, RNA-dependent RNA polymerase inhibitor

## Abstract

Favipiravir, an oral antiviral medication, has been approved in Japan for the treatment of patients with novel or re-emerging influenza and severe fever with thrombocytopenia syndrome (SFTS). Randomized, double-blind, placebo-controlled trials were conducted in an East Asian population to characterize the pharmacokinetics and tolerability of an injectable favipiravir formulation. In the single-ascending dose trial, subjects received a single intravenous (IV) dose of 300–2,400 mg of favipiravir. In the multiple dose trial, 1,800 mg was administered twice daily on the first day, followed by 800 mg twice daily for the subsequent 10 days, which is the approved tablet dose for SFTS in Japan. This multiple-dose trial was divided into a cohort receiving intravenous administration throughout the treatment period (IV cohort) and a cohort switching to oral administration from Day 6 (Switching cohort). After a single administration of ≤2,400 mg, the pharmacokinetic parameters increased in a dose-dependent manner but showed slight deviation from linearity. The geometric mean maximum plasma concentration following a single 2,400 mg dose was 126 (range: 110 to 138) μg/mL. In the IV cohort, the trough (at 12 h post-dose) plasma concentrations were maintained at approximately 85 μg/mL after Day 2. These concentrations were approximately 20% higher than those achieved with the same oral doses. In the Switching cohort, these concentrations were maintained throughout the treatment. No serious adverse events were observed. These findings support advancing injectable favipiravir into clinical evaluation.

**CLINICAL TRIALS:**

This study is registered with the Japan Registry of Clinical Trials as jRCT2071210042 and jRCT2071210126.

## INTRODUCTION

Favipiravir is an oral antiviral drug developed by Toyama Chemical (now Fujifilm Toyama Chemical) and was approved in Japan in 2014 for treatment of novel or re-emerging influenza virus infections in which other antiviral drugs are ineffective or insufficiently effective (1) and in 2024 for treatment of severe fever with thrombocytopenia syndrome (SFTS) virus infections (2). Favipiravir exerts its effects by selectively inhibiting viral RNA-dependent RNA polymerase after conversion to its active ribosyl triphosphate form by intracellular enzymes (3). Its primary mechanism of action involves a combination of chain termination and lethal mutagenesis (4–6). Therefore, favipiravir may be effective against a wide range of RNA viruses. In fact, the *in vitro* 50% inhibitory concentrations (IC_50_) of favipiravir against seasonal influenza viruses, SFTS virus, Crimean-Congo hemorrhagic fever virus, avian influenza (A/H5N1) virus, Rift Valley fever virus, and Ebola virus are 0.01–0.48 (7), 0.9 (8), 1.1 (9), 0.4–1.9 (10), 5.0 (11), and 10.5 μg/mL (12), respectively. Furthermore, the efficacy of favipiravir has also been suggested in these RNA virus infection models (7–10, 12, 13).

Favipiravir irreversibly inhibits its main metabolic enzyme, aldehyde oxidase (AO), in a concentration-dependent manner (mechanism-based inhibition, MBI). Thus, multiple administrations of favipiravir result in plasma concentrations that exceed those expected after a single administration (1). This characteristic makes it possible to maintain the favipiravir plasma concentrations within a certain range by administering a high loading dose on the first day, followed by a maintenance dose.

In some viral infections, such as Crimean-Congo hemorrhagic fever, SFTS, or severe influenza, oral administration may be difficult due to increased aspiration risk associated with patient disorientation. Furthermore, since these diseases often cause gastrointestinal symptoms, such as vomiting and diarrhea, drug absorption after oral administration may be impaired. Therefore, an injectable formulation that can reliably deliver favipiravir into the plasma is desirable for bedside use (14). After development of an injectable formulation of favipiravir, we planned a Phase I study to evaluate its safety, tolerability, and pharmacokinetics in healthy, young East Asian adult males.

## MATERIALS AND METHODS

This Phase 1 study consisted of two trials: a single-ascending dose trial and a multiple-dose trial.

### Overview and design

Both trials had a randomized, double-blind, and placebo-controlled design. In a clinical trial conducted on patients with SFTS virus infection, favipiravir treatment reduced the fatality risk by half when patients received 1,800 mg twice daily on Day 1 as a loading dose, followed by 800 mg twice daily as a maintenance dose (1,800/800 mg twice daily) for 9 days (15). Based on these findings, we set the investigational dose for intravenous (IV) administration at a level that achieves plasma concentrations comparable to, rather than exceeding, those obtained via oral administration. In healthy East Asian subjects, a single 2,400 mg dose of favipiravir (supra-therapeutic dose) raised no tolerability concerns, with a maximum plasma concentration (C_max_) of 155 μg/mL (16). A dose of 300 mg was selected as the initial dose for the single-ascending dose trial. This dose was selected because, even if the C_max_ increased 10-fold compared to the plasma concentration expected after a 300 mg oral dose, it would not exceed 155 μg/mL. The infusion time was set to 60 min, corresponding to the time required to reach the maximum plasma concentration following oral administration (t_max_). For subsequent cohorts, up to four cohorts within a dose of ≤2,400 mg were selected according to the prespecified protocol shown in Fig. S1 of the Supplemental material. If the pharmacokinetics of favipiravir in the single-ascending dose trial did not differ significantly from those observed after oral administration, 1,800/800 mg twice daily established as the approved oral dose in Japan was selected as the investigational dose for the multiple-dose trial (2).

In cohort 1 of the multiple-dose trial (IV cohort), a regimen of 1,800/800 mg twice daily was administered intravenously for 11 days. In cohort 2 (Switching cohort), the same dosage in the IV cohort was administered intravenously on Days 1–5, followed by a switch to oral administration on Days 6–11.

The single-ascending dose trial enrolled 10 healthy subjects each in up to five cohorts. The subjects were randomized in a 4:1 ratio, with eight subjects in each cohort receiving favipiravir and two subjects receiving a matching placebo. In the multiple-dose trial, 24 healthy subjects (12 per cohort) were enrolled and randomly assigned in a 3:1 ratio; in each cohort, nine subjects received favipiravir, and three subjects received a matching placebo.

An increase in blood uric acid levels following favipiravir administration is a drug-specific event, which may compromise study blinding (17). Therefore, in this multiple-dose trial, designated unblinded staff monitored blood uric acid levels after administration of the investigational drug, and these results were withheld from investigators and other study personnel to maintain blinding.

### Participants

Healthy East Asian males at birth aged 20–39 years with a body weight of ≥55 kg and a body mass index ranging from 18.5 to 29.9 kg/m^2^ were eligible to participate in the study. Potential subjects were screened within 28 days before the first dose. Subjects were excluded if they had a history of smoking, including any nicotine-containing products within 90 days before dosing, to avoid potential effects of nicotine on the autonomic nervous system; had previously received favipiravir; had an abnormal corrected QT interval using Fridericia’s formula (>450 msec) or PR interval (>210 msec) on electrocardiograms (ECGs); or had been diagnosed with hypouricemia (<1 mg/dL) or xanthine urinary calculus. Subjects were required to use condoms, alone or in combination with barrier methods (e.g., diaphragms), and to abstain from sexual intercourse with pregnant women from dosing initiation until 7 days after the end of dosing.

### Study procedure

For pharmacokinetic measurements, 4 mL of blood was drawn from each sampling time point using vacuum blood collection tubes containing sodium heparin. In the single-ascending dose trial, blood was sampled at 12 time points, with the initiation of dosing defined as 0 h: once before, during, and at the end of dosing, and at nine time points up to 57 h post-dose. In the multiple-dose trial, blood was sampled at 45 time points, with the initiation of the morning dose on each administration day defined as 0 h: once before, during, and at the end of the morning dose (60 min after the initiation); at five time points up to 12 h post-dose on Days 1 and 6; once before, during, and at the end of the morning dose; and at eight time points up to 72 h post-dose on Day 11. During all other administration periods, the sampling was performed before and at the end of the morning dose. Urine samples were collected using cups and pooled into the designated polypropylene containers at each collection interval. Complete voiding was ensured before the initial collection on Day 1 and at the end of each collection interval. The sampling time points for each trial are presented in Tables S1 to S3 of the Supplemental material.

Bioanalytical samples were shipped on dry ice and stored at −20°C in the analytical facility (Mediford, Tokyo, Japan). Plasma and urine concentrations of favipiravir and its metabolite M1 were measured using a validated high-performance liquid chromatography (HPLC) method. Chromatographic separation was performed on an Inertsil ODS-3 column (4.6 mm I.D. ×100 mm, 3 µm; GL Sciences, Tokyo, Japan) maintained at 30°C. Favipiravir and M1 were detected via UV absorption at 360 nm using a Shimadzu LC-20AD pump coupled to a Shimadzu SPD-20A UV detector (Shimadzu, Kyoto, Japan). The calibration curves for both analytes ranged from 0.0200 to 20.0 µg/mL. Samples with analyte concentrations exceeding, or expected to exceed, this range were appropriately diluted before analysis. The lower limit of quantitation (LLOQ) was 0.0200 µg/mL; results below the LLOQ were reported as below the limit of quantitation (BLOQ).

Treatment-emergent adverse events (TEAEs) were recorded up to the final study visit. In the multiple-dose trial, changes in blood uric acid levels were not disclosed until unblinding; therefore, these changes were not considered TEAEs.

### Preparation of injectable formulation

The favipiravir formulation used in this study was a lyophilized powder containing additives such as sodium hydroxide and hydrochloric acid, stored at room temperature under light-protected conditions. Each vial containing 600 mg of favipiravir was dissolved in 10 mL of sterile water for injection. An aliquot corresponding to the required dose was taken and diluted with physiological saline to a final volume of 100 mL for doses ≤ 1,200 mg or 200 mL for doses > 1,200 mg. The dosing solutions of the investigational drug were prepared within 3 h prior to administration. For the placebo, an equivalent volume of physiological saline was used.

### Single-ascending dose trial

Subjects were quarantined in the medical institution from the morning of Day −1, before the day of administration, until completion of the 57 h post-dose. Vital sign measurements, ECGs, and physical examinations were performed on Day 8 in addition to daily assessments during the confinement period. Clinical laboratory tests (serum chemistry, hematology, and urinalysis) were performed on Days −1, 2, 3, and 8.

### Multiple-dose trial

Subjects were quarantined in the medical institution from the morning of Day −1 until 72 h after the last dose. Vital sign measurements and physical examinations were performed daily during the confinement period and on Day 20. ECGs were performed on Days −1, 1, 2, 5, 6, 8, 11, and 20. Clinical laboratory tests were measured on Days −1, 2, 5, 8, 11, 14, and 20. In the IV cohort, the three cases of nail discoloration were first noted 2 days (Day 13), 3 days (Day 14), and 8 days (Day 19) after the end of dosing, respectively; therefore, in the Switching cohort, nail and skin discolorations were assessed on Days −1, 12, 20, and 41.

### Study outcomes and statistical methods

The plasma pharmacokinetic parameters such as C_max_, t_max_, area under the drug concentration–time curve from time 0 to the last measurable time (AUC_t_), AUC from time 0 to infinity (AUC_inf_), AUC during a dosage interval (AUC_τ_), apparent total body clearance of the drug from plasma (CL), elimination rate constant from the central compartment (k_e_), apparent volume of distribution based on the AUC (V_d_), elimination half-life (t_1/2_), and mean residence time (MRT) were calculated from the overall plasma concentration-versus-time profiles by non-compartmental methods. Additionally, the natural logarithm (ln)-transformed C_max_, AUC_t_, and AUC_inf_ were presented for favipiravir and M1. Renal clearance (CL_R_) was calculated for favipiravir and M1. The percentage of the dose excreted in urine (%f_e_) was also calculated for favipiravir following each dose regimen. The %f_e_ of M1 was calculated based on its molecular weight for each dosing regimen.

Because favipiravir pharmacokinetics are assumed to exhibit high variability, geometric means were used to summarize C_max_ and AUC values, while arithmetic means were used for other pharmacokinetic parameters.

Dose proportionality was evaluated in the single-ascending trial using a linear regression model over the 300–2,400 mg dose range. The model was used to calculate the slope and its 95% confidence intervals (*CIs*) for the logarithm-transformed C_max_ and AUC_inf_. Dose proportionality was to be concluded if the 95% CI spanned 1.0. Multiple-dose simulations using the physiologically based pharmacokinetic model of MBI of the main metabolic enzyme, AO (MBI-PK model), were performed (18), and the results were compared with observed concentrations. This was a two-compartment model (C_p_ and E_act_) with a first-order input from the dosing compartment (X1). C_p_ is the central compartment, and E_act_ is the enzyme compartment. The model assumes that CL (CL = V_max_/[K_m_ +C_p_]) changes with the central compartment concentration (C_p_ = X2/V), as shown in Fig. S2 of the Supplemental material. Daily C_max_, minimum plasma concentration (C_min_), and AUC for the treatment period were simulated using the MBI-PK model.

Safety was assessed based on TEAEs, including clinical laboratory tests, vital sign measurements, ECGs, physical examination findings, and skin and nail discoloration. The incidence of TEAEs was defined as the proportion of patients who experienced at least one TEAE.

To calculate summary statistics, the BLOQ values were treated as 0. All analyses and summaries were calculated using Phoenix WinNonlin version 8.2 (Certara, St. Louis, MO, USA) and SAS Version 9.4 (SAS Institute, Cary, NC, USA).

## RESULTS

### Participant disposition and demography

In the single-ascending dose trial, 50 eligible subjects, 10 from each cohort, who confirmed their participation on Day −1, were enrolled. All subjects received the investigational drug (favipiravir or matching placebo) to which they were assigned. No participants discontinued the trial.

In the multiple-dose trial, 24 eligible subjects, 12 from each cohort, who confirmed their participation on Day −1, were enrolled. All subjects received the investigational drug to which they were assigned. Only one subject in the Switching cohort discontinued treatment due to TEAEs.

The baseline demographics and clinical characteristics across treatments in each trial are summarized in Table 1. All subjects were of East Asian ethnicity, specifically of Japanese origin, and male at birth. There were no notable differences in the subject demographics between the dose regimens.

**TABLE 1:** Demographics and baseline characteristics of healthy subjects in the study

Characteristics		Single-ascending dose						Multiple dose		
		Placebo		Favipiravir				Placebo	Favipiravir 1,800/800 mg^*a*^	
			300 mg	600 mg	1,200 mg	1,800 mg	2,400 mg		IV^*b*^	IV to oral^*c*^
		(*n* = 10)	(*n* = 8)	(*n* = 8)	(*n* = 8)	(*n* = 8)	(*n* = 8)	(*n* = 6)	(*n* = 9)	(*n* = 9)
Sex^***d***^**,** n (%)	Male	10 (100)	8 (100)	8 (100)	8 (100)	8 (100)	8 (100)	6 (100)	9 (100)	9 (100)
Race, n (%)	Asian	10 (100)	8 (100)	8 (100)	8 (100)	8 (100)	8 (100)	6 (100)	9 (100)	9 (100)
Age, years	Median	22.5	24.5	25.5	27.0	29.0	21.5	28.5	27.0	27.0
	Range	21–35	20–33	21–39	21–31	21–39	20–25	25–35	20–38	21–38
Weight, kg	Median	60.9	63.3	63.3	69.2	60.7	63.5	66.9	66.3	64.2
	Range	56.9–68.6	58.7–72.8	60.3–69.4	57.8–75.7	57.4–66.9	57.2–70.8	59.9–72.9	57.1–78.1	56.5–77.6
BMI^*e*^, kg/m2	Median	20.3	22.5	20.6	22.5	20.3	21.8	22.4	22.2	21.8
	Range	19.0–23.5	21.1–23.8	18.9–23.5	19.1–24.8	19.6–22.0	19.4–22.9	19.9–25.3	19.5–25.1	19.7–24.8
CL_CR_, mL/min	Median	110.6	116.6	110	112.3	98.5	119	112.7	111.4	105.1
	Range	85.5–145.4	102.8–122.0	82.2–127.8	107.6–130.6	86.6–128.7	113.3–143.1	108.7–121.0	85.8–157.5	73.5–137.4

^*a*^ 1,800 mg twice daily on the first day, followed by 800 mg twice daily for the subsequent 10 days.

^*b*^ 60-min intravenous administration throughout the treatment period.

^*c*^ During the treatment period, intravenous administration for the first 5 days, followed by a switch to oral administration.

^*d*^ Sex at birth.

^*e*^ Body mass index.

### Pharmacokinetics

#### Single administration

**Fig 1:**
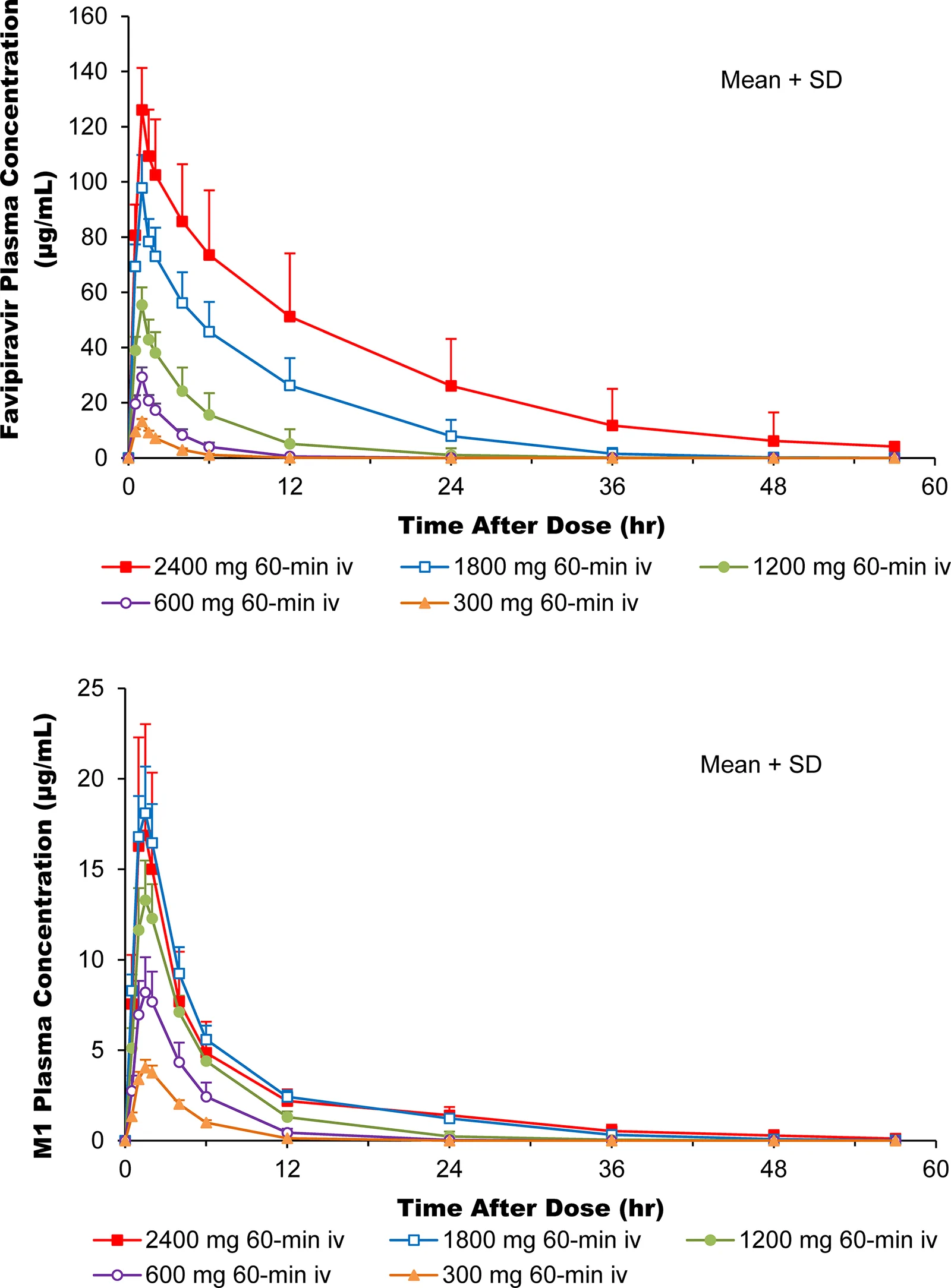
Time course of the plasma concentrations of favipiravir (top) and the main metabolite M1 (bottom) over 57 hours after five intravenous doses.

**Fig 2:**
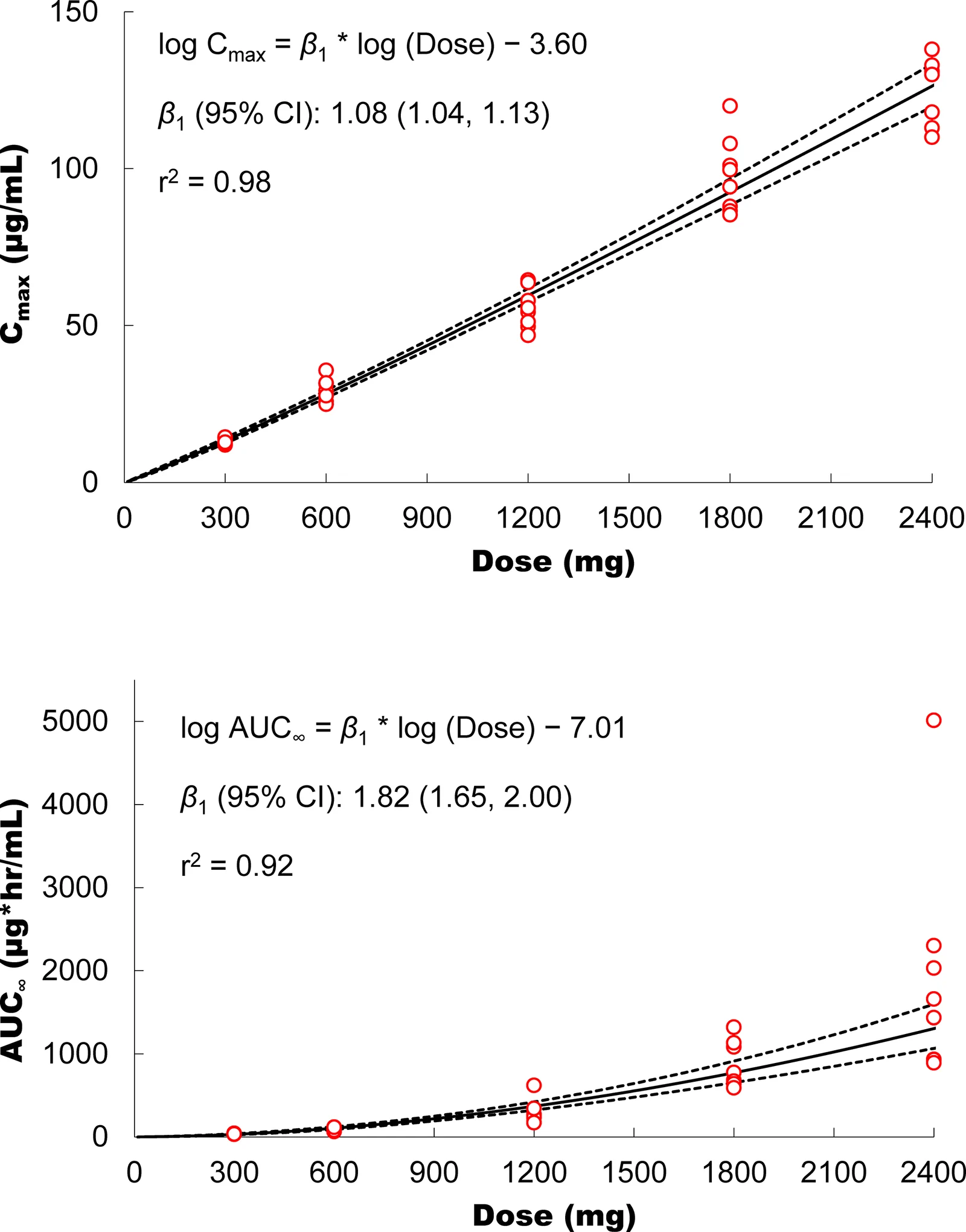
Scatter plots with regression lines show C_max_ (top) and AUC_inf_ (bottom) increasing with dose from 300 to 2,400 mg. C_max_ scales nearly linearly with slope 1.08, while AUC_inf_ shows supra-linear increase with slope 1.82.

According to the prespecified protocol, subjects in all five cohorts received a 60-min intravenous administration. The plasma concentration of favipiravir increased in a monophasic and dose-dependent manner. The mean concentrations after 57 h at doses ≤ 1,800 mg were almost BLOQ and at 2,400 mg were 4.10 μg/mL (Fig. 1). M1 concentrations also increased with the same trend as favipiravir, with the mean concentrations after 57 h at doses ≤ 1,800 mg being almost BLOQ and at 2,400 mg being 0.11 μg/mL (Fig. 1). The C_max_ and AUC_inf_ increased in a dose-dependent manner. However, when assessed using the power model, both lower limits of the 95% *CIs* exceeded 1.0; consequently, linearity could not be confirmed. The degree of divergence in the C_max_ was slight (Fig. 2).

After a single intravenous administration of favipiravir at 2,400 mg, the geometric mean C_max_ and the AUC_inf_ reached 126 (range: 110 to 138) μg/mL and 1,600 (range: 892 to 5,013) μg·h/mL, respectively. The percentage coefficient of variation (CV%) for C_max_ did not change across doses, but that for AUC_inf_ ranged from 11% to 72%, which was large at 2,400 mg. The mean t_1/2_ ranged from 1.5 to 7.3 h and was dose-dependent. The CV% at the 2,400 mg dose was as high as 111%, indicating extremely large variability. The mean V_d_ was approximately 19 L at ≤1,200 mg, decreasing to approximately 13 L at 1,800 mg and 2,400 mg. Similarly, the mean CL decreased with increasing dose. The mean CL_R_ was 0.03–0.05 L/h, which was lower than CL. The median t_max_ of M1 was 1.5 h regardless of the dosage. The geometric mean C_max_ of M1 reached 14.8 (range: 2.9 to 22.3) μg/mL at 2,400 mg. The mean t_1/2_ of M1 increased in a dose-dependent manner and was 2.0 h at 300 mg and 13 h at 2,400 mg. The mean ratio for AUC_inf_ of M1 to AUC_inf_ of favipiravir was 0.5 at 300 mg and decreased with increasing dose to 0.1 at 2,400 mg (Table 2).

**TABLE 2:** Pharmacokinetic parameters following a single 60-min intravenous administration of favipiravir

Compound	Parameter^*a*^	300 mg	600 mg	1,200 mg	1,800 mg	2,400 mg
		(***n*** = 8)	(*n* = 8)	(*n* = 8)	(***n*** = 8)	(*n* = 8)
Favipiravir	C_max_ (μg/mL)	13.20 (7.0)	29.12 (12.1)	55.13 (11.5)	97.21 (12.2)	125.94 (8.8)
		11.9, 14.4	24.9, 35.7	46.9, 64.5	85.3, 120	110, 138
	C_24_ (μg/mL)**^*b*^**	0.00 (113.3)	0.01 (115.6)	0.20 (238.4)	6.20 (74.8)	18.17 (87.8)
		0.00, 0.00	0.00, 0.05	0.04, 7.07	2.77, 18.20	5.96, 73.10
	t_max_ (h)	1.0 (0.0)	1.0 (0.0)	1.0 (1.8)	1.0 (0.0)	1.1 (16.6)
		1.0, 1.0	1.0, 1.0	1.0, 1.05	1.0, 1.0	1.0, 1.5
	AUC_inf_ (μg·h/mL)	35.52 (11.4)	91.76 (20.8)	264.43 (50.3)	826.25 (32.0)	1,600.87 (71.9)
		30.50, 43.30	65.18, 117.42	171.22, 619.62	589.94, 1,319.87	891.77, 5,012.60
	AUC_t_ (μg·h/mL)	35.32 (11.1)	91.13 (21.1)	264.08 (50.4)	825.65 (32.0)	1,546.25 (56.9)
		30.41, 42.83	64.91, 117.23	171.08, 619.44	589.79, 1,318.55	891.61, 3,847.85
	t_1/2_ (h)	1.5 (9.4)	2.0 (20.8)	2.9 (17.3)	3.9 (15.0)	7.3 (111.2)
		1.4, 1.8	1.5, 2.6	2.3, 3.9	3.2, 4.7	3.3, 27.2
	V_d_ (L)	18.79 (7.7)	18.52 (7.5)	19.17 (22.3)	12.82 (32.4)	12.50 (36.0)
		16.93, 21.78	15.97, 20.05	10.92, 23.86	8.00, 18.20	7.75, 18.78
	MRT (h)	2.0 (9.8)	2.6 (21.7)	4.4 (43.7)	8.0 (22.0)	13.7 (76.1)
		1.8, 2.4	1.9, 3.5	3.0, 9.1	6.3, 11.2	7.2, 38.6
	CL (L/h)	8.49 (10.8)	6.67 (21.5)	4.84 (34.3)	2.26 (28.2)	1.17 (48.4)
		6.93, 9.84	5.11, 9.20	1.94, 7.01	1.36, 3.05	0.48, 2.69
	CL_R_ (L/h)	0.05 (33.2)	0.03 (23.7)	0.03 (28.4)	0.04 (14.2)	0.04 (18.3)
		0.04, 0.09	0.02, 0.05	0.02, 0.05	0.03, 0.04	0.03, 0.05
	%fe (%)^*c*^	0.6 (35.5)	0.5 (24.4)	0.7 (30.4)	1.7 (35.4)	2.7 (51.8)
		0.4, 1.0	0.3, 0.7	0.4, 1.1	1.1, 2.7	1.5, 5.7
M1	C_max_ (μg/mL)	4.02 (10.7)	8.02 (23.6)	13.09 (16.6)	18.02 (14.1)	14.83 (36.4)
		3.67, 4.96	6.02, 11.7	8.30, 15.4	14.9, 22.1	2.92, 22.3
	C_24_ (μg/mL)**^*b*^**	0.00 (62.2)	0.02 (78.8)	0.16 (120.3)	1.18 (29.4)	1.31 (33.0)
		0.00, 0.01	0.00, 0.09	0.06, 0.90	0.69, 1.71	0.51, 1.93
	t_max_ (h)	1.5 (0.0)	1.5 (0.0)	1.5 (0.0)	1.4 (12.3)	1.5 (0.0)
		1.5, 1.5	1.5, 1.5	1.5, 1.5	1.0, 1.5	1.5, 1.5
	AUC_inf_ (μg·h/mL)	17.82 (10.0)	40.09 (25.7)	76.95 (8.9)	124.12 (9.0)	121.46 (21.2)
		15.59, 21.13	30.95, 64.80	65.79, 87.25	111.26, 146.22	62.94, 144.91
	AUC_inf_ ratio**^*d*^**	0.506 (14.2)	0.448 (24.3)	0.307 (29.1)	0.157 (30.3)	0.093 (53.2)
		0.394, 0.581	0.326, 0.599	0.121, 0.415	0.092, 0.218	0.013, 0.162
	AUC_t_ (μg·h/mL)	17.44 (9.9)	39.79 (25.9)	76.44 (8.8)	123.72 (9.2)	112.81 (28.4)
		15.28, 20.75	30.31, 64.42	65.49, 85.98	110.00, 146.05	38.10, 144.72
	t_1/2_ (h)	2.0 (7.9)	2.7 (18.5)	3.9 (27.9)	5.2 (15.2)	12.8 (117.3)
		1.8, 2.3	1.8, 3.1	2.9, 6.4	4.4, 6.5	4.4, 48.5
	MRT (h)	3.1 (7.0)	3.8 (17.7)	5.2 (37.4)	8.2 (23.8)	16.9 (108.8)
		2.8, 3.5	2.7, 4.6	4.0, 10.0	6.3, 11.6	7.1, 61.4
	CL_R_ (L/h)	15.99 (13.2)	14.30 (20.7)	13.52 (14.6)	11.90 (10.6)	12.79 (22.8)
		13.05, 18.52	8.83, 18.33	11.13, 16.80	10.29, 14.18	7.57, 16.59
	%fe (%)**^*c*^**	85.7 (6.0)	85.0 (3.7)	78.3 (9.9)	74.3 (6.0)	61.7 (30.7)
		75.4, 90.1	80.5, 88.9	63.4, 85.6	67.7, 79.6	18.0, 80.0

^*a*^ For each parameter, the upper row represents the mean value (coefficient of variation), while the lower row represents the corresponding minimum and maximum values. Among these parameters, C_max_ and AUCs are presented as geometric means, whereas all other pharmacokinetic parameters are expressed as arithmetic means.

^*b*^ Plasma concentrations at 24 h post-dose.

^*c*^ Fraction of the administered favipiravir dose excreted in urine up to 57 h post-dose.

^*d*^ AUC_inf_ of M1 to AUC_inf_ of favipiravir ratio.

Following a single intravenous administration of favipiravir at ≤2,400 mg, the cumulative %f_e_ at 57 h was low, ranging from 0.5% to 2.7%. In contrast, M1 values were high, ranging from 62% to 86%; most of the favipiravir was excreted as M1 in the urine. The cumulative %f_e_ of favipiravir and M1 ranged from 64% to 86%, decreasing with dose and by 26% at 2,400 mg compared with 300 mg.

#### Multiple administrations

**Fig 3:**
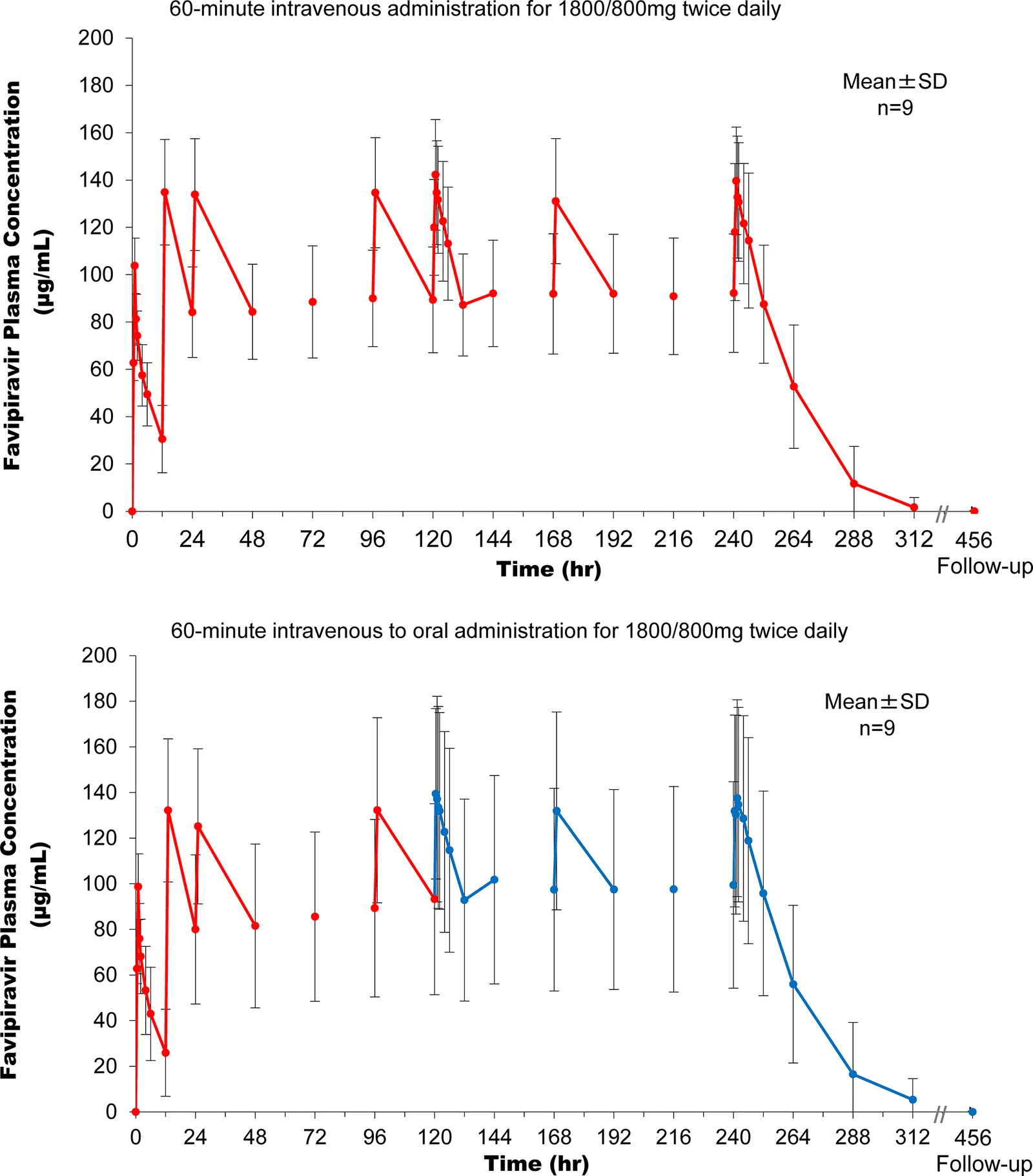
The dosing interval was 12 h. The plasma concentration profiles of favipiravir in both dosing regimens were comparable, demonstrating consistent exposure throughout the treatment period.

The plasma concentration of favipiravir in the IV cohort after the second administration on Day 1 was similar throughout the administration period (Fig. 3). The geometric mean of C_max_ and AUC were 100 μg/mL and 1,000 μg·h/mL (AUC_inf_) on Day 1 and 140 μg/mL and 1,300 μg·h/mL (AUC_τ_) on Days 6 and 11, respectively. In contrast, the M1 plasma concentration decreased after the second administration on Day 1 compared with that at the first administration on Day 1, and the geometric mean of C_max_ was 14.0 μg/mL on Day 1 and 3.1–3.3 μg/mL on Days 6 and 11. The t_1/2_ of favipiravir did not change on Days 1 and 11, but that of M1 was prolonged by approximately 4-fold on Day 11 compared to Day 1 (Table 3).

**TABLE 3:** Pharmacokinetic parameters following multiple administration of favipiravir^*h*^

		1,800 mg/800 mg twice daily^*a*^					
Compound	Parameter^*b*^	IV iIntravenous administration)^*c*^			Switching (IV to oral) administration^*d*^		
		Day 1 (*n* = 9)	Day 6 (*n* = 9)	Day 11 (*n* = 9)	Day 1 (*n* = 9)	Day 6 (*n* = 8)	Day 11 (*n* = 8)
Favipiravir	C_max_ (μg/mL)	103.19 (11.3)	141.20 (16.4)	139.51 (17.0)	97.79 (14.7)	138.51 (28.6)	139.46 (28.9)
		85.7, 122	119, 191	113, 181	77.2, 126	83.5, 199	97.1, 220
	C_trough_ (μg/mL)^*e*^	28.23 (46.6)	85.10 (24.7)	84.53 (28.5)	18.92 (73.5)	82.85 (47.6)	87.00 (46.8)
		17.7, 63.0	58.8, 135	51.2, 138	4.11, 58.0	36.3, 158	45.9, 164
	t_max_ (h)	1.0 (0.0)	1.1 (19.8)	1.0 (25.0)	1.0 (0.0)	0.6 (37.0)	1.4 (88.52)
		1.0, 1.0	1.0, 1.5	0.5, 1.5	1.0, 1.0	0.5, 1.0	0.5, 4.0
	AUC^*f*^(μg·h/mL)	968.03 (53.6)	1,320.04 (20.4)	1,314.87 (23.3)	766.37 (68.2)	1,279.35 (38.1)	1,329.57 (37.5)
		623.58, 2,449.50	1,018.56, 1,926.60	916.35, 1,976.75	295.36, 2,075.01	669.29, 2,114.89	789.75, 2,241.25
	AUC_t_ (μg·h/mL)	601.88 (23.2)	1,305.59 (20.4)	2,904.50 (42.6)	518.35 (38.0)	1,265.24 (38.0)	2,975.97 (58.9)
		479.42, 942.30	1,008.60, 1,903.76	1553.13, 6,084.35	278.34, 915.22	663.15, 2,088.11	1,421.95, 6,773.65
	AUC_t_ / AUC_inf_	0.635 (20.5)	0.404 (14.3)	0.993 (1.8)	0.702 (27.3)	0.385 (33.1)	0.976 (4.4)
		0.385, 0.786	0.323, 0.512	0.945, 1.000	0.427, 0.942	0.205, 0.556	0.896, 1.000
	t_1/2_ (h)	8.7 (40.2)	16.0 (17.6)	8.6 (52.4)	7.5 (56.8)	19.1 (46.2)	10.5 (68.4)
		5.3, 16.6	11.3, 20.5	5.5, 19.4	2.9, 14.4	10.1, 35.5	5.6, 22.4
	V_d_ (L)	22.13 (11.3)	5.65 (15.7)	3.17 (23.8)	22.15 (11.1)	6.12 (35.0)	3.51 (30.0)
		17.58, 25.24	4.01, 6.64	2.44, 4.49	17.35, 25.24	4.02, 9.78	2.06, 5.25
	MRT (h)	12.0 (43.1)	22.7 (18.2)	15.2 (31.3)	10.3 (60.5)	27.6 (46.2)	17.7 (45.5)
		7.1, 23.6	15.9, 29.3	10.2, 26.1	3.7, 20.5	14.5, 51.3	11.5, 30.9
	CL (L/h)	1.99 (34.0)	0.26 (29.3)	0.29 (37.9)	2.86 (64.5)	0.29 (72.4)	0.31 (53.8)
		0.73, 2.89	0.14, 0.41	0.12, 0.51	0.87, 6.09	0.08, 0.67	0.11, 0.56
	%fe^*g*^ (%)	1.2 (25.7)	2.7 (31.7)	3.4 (30.2)	1.0 (44.2)	2.5 (49.9)	3.2 (51.4)
		0.6, 1.5	1.6, 4.0	2.1, 5.1	0.3, 1.5	0.9, 3.9	2.9, 11.9
M1	C_max_ (μg/mL)	13.87 (30.3)	3.09 (18.3)	3.33 (22.7)	17.07 (28.8)	3.19 (25.5)	3.14 (30.4)
		6.14, 22.0	2.24, 4.16	1.90, 4.43	6.71, 23.9	1.98, 4.57	1.58, 4.64
	C_trough_ (μg/mL)^*e*^	2.19 (25.3)	2.63 (16.3)	2.99 (20.2)	2.06 (36.5)	2.73 (26.6)	2.84 (29.4)
		1.03, 3.00	1.78, 3.30	1.60, 3.75	1.17, 3.21	1.87, 3.72	1.58, 4.02
	t_max_ (h)	1.5 (0.0)	2.3 (89.6)	1.9 (82.8)	1.5 (0.0)	0.8 (31.8)	4.1 (120.6)
		1.5, 1.5	1.0, 6.0	0.5, 6.0	1.5, 1.5	0.5, 1.0	0.5. 12.0
	AUC^*f*^ (μg·h/mL)	87.12 (27.3)	32.08 (20.9)	37.23 (21.6)	95.08 (27.4)	32.78 (25.3)	35.03 (29.1)
		38.81, 130.31	23.73, 44.03	20.95, 48.96	43.85, 138.65	22.64, 46.00	18.71, 49.46
	AUC_t_ (μg·h/mL)	72.37 (29.3)	33.17 (17.3)	130.75 (16.1)	82.54 (28.2)	33.53 (24.8)	119.79 (47.9)
		30.80, 113.16	23.43, 43.47	104.06, 156.63	34.88, 121.15	22.33, 45.38	71.27, 250.16
	AUC_t_ / AUC_inf_	0.831 (3.9)	0.122 (60.2)	0.924 (18.2)	0.870 (7.0)	0.101 (64.7)	0.892 (21.2)
		0.794, 0.874	0.064, 0.221	0.477, 0.995	0.756, 0.944	0.021, 0.182	0.458, 0.996
	AUC_τ_ ratio	0.132 (37.7)	0.026 (39.1)	0.030 (33.8)	0.184 (48.4)	0.029 (40.2)	0.029 (40.8)
		0.032, 0.208	0.012, 0.043	0.011, 0.048	0.043, 0.291	0.012, 0.043	0.009, 0.047
	t_1/2_ (h)	4.6 (11.8)	81.1 (48.5)	19.8 (122.2)	3.9 (25.3)	132.1 (93.4)	23.1 (121.6)
		3.9, 5.4	32.1, 122.1	8.5, 83.5	2.8, 5.7	39.6, 389.2	8.2, 89.5
	MRT (h)	6.1 (11.4)	116.6 (48.6)	32.7 (95.8)	5.3 (25.0)	190.6 (93.4)	37.2 (97.6)
		5.1, 6.9	46.1, 175.9	16.0, 115.5	3.7, 7.8	57.5, 561.7	16.8, 123.8
	%fe^*g*^ (%)	37.5 (22.1)	41.1 (16.3)	46.7 (16.9)	47.4 (27.1)	49.6 (23.0)	55.2 (18.1)
		19.2, 46.5	26.0, 48.4	27.8, 54.8	29.1, 68.3	36.5, 64.3	43.0, 68.2

^*a*^ 1,800 mg twice daily on the first day, followed by 800 mg twice daily for the next 10 days.

^*b*^ For each parameter, the upper row represents the mean value (coefficient of variation), while the lower row represents the corresponding minimum and maximum values. Among these parameters, C_max_ and AUCs are presented as geometric means, whereas all other pharmacokinetic parameters are expressed as arithmetic means.

^*c*^ 60-min intravenous administration throughout the treatment period.

^*d*^ During the treatment period, favipiravir was administered intravenously for the first 5 days, followed by a switch to oral administration.

^*e*^ Plasma concentrations at 12 h post-dose.

^*f*^ AUC_inf_ on Day 1; AUC_τ_ on Days 6 and 11 are illustrated.

^*g*^ Fraction of the cumulative amount excreted in urine from the initiation of administration up to 72 h after the last administration relative to the total administered dose over 11 days (18,800 mg).

^*h*^ The AUC_t_ on Days 1 and 6 was calculated using plasma concentrations measured up to 12 h post-dosing, and the AUC_inf_ on Day 11 was calculated using plasma concentrations measured up to 72 h post-dosing. AUC_inf_ was extrapolated from the last measurement time (12 h on Days 1 and 6; 72 h on Day 11) using the terminal elimination rate constant. AUC_τ_ was calculated over the dosing interval (12 h).

In the Switching cohort, one subject discontinued the treatment on Day 5 due to a TEAE (visual impairment); therefore, pharmacokinetic data from Day 6 onward were excluded only for this subject. The plasma concentration of favipiravir in this cohort increased after the second administration on Day 1 and remained elevated throughout the intravenous-dosing period for 5 days. Thereafter, when the drug was switched to oral administration from Day 6, there was a slight increase compared to intravenous administration; however, no significant impact was observed owing to the alternate administration method. (Fig. 3). The geometric mean of C_max_ of favipiravir was similar to that of the IV cohort, but the geometric mean of AUC_τ_ on Days 1 and 6 was slightly lower than that of the IV cohort and tended to increase with the number of doses. Throughout the dosing period, the plasma concentration of M1 was similar to that in the IV cohort. There was a 6-fold increase in the t_1/2_ of M1 on Day 11 compared to that on Day 1, and the prolongation was greater than that in the IV cohort. In both cohorts, the cumulative %f_e_ of favipiravir and M1 from the initiation of dosing to 72 h after the last dose was 50%–60% (Table 3).

#### Pharmacokinetic simulation of favipiravir

The plasma concentrations of favipiravir after multiple doses were simulated using the MBI-PK model. The estimated concentration curves of favipiravir predicted by this model generally corresponded to the measured values in both cohorts (Fig. S3 of the Supplemental material. The C_max_ and C_min_ of favipiravir in the IV cohort ranged from 130 to 135 μg/mL and from 83 to 88 μg/mL, respectively, after the second dose on Day 1 throughout the treatment period. Similarly, the C_max_ and C_min_ of favipiravir in the Switching cohort, which switched to oral administration from Day 6, ranged from 120 to 138 μg/mL and from 75 to 85 μg/mL, respectively.

### Safety

No serious or severe TEAEs were reported in either trial (Table 4).

**TABLE 4:** Summary of treatment-emergent adverse events

Characteristics	Single-ascending dose						Multiple dose		
	Placebo		Favipiravir				Placebo	Favipiravir 1,800/800 mg^*a*^	
Adverse events (AEs)		300 mg	600 mg	1,200 mg	1,800 mg	2,400 mg		IV^*b*^	IV to oral^*c*^
	(*n* = 10)	(*n* = 8)	(*n* = 8)	(*n* = 8)	(*n* = 8)	(*n* = 8)	(*n* = 6)	(*n* = 9)	(*n* = 9)
Overall, n (nE)	1 (1)				1 (1)	2 (5)	2 (3)	5 (7)	7 (10)
Mild	1 (1)				1 (1)	2 (4)	2 (3)	5 (7)	7 (8)
Moderate						1 (1)			1 (2)
Severe									
Serious AEs, n (nE)									
Terms of AEs, n (nE)									
Visual impairment									1 (1)
Diarrhea							2 (3)		2 (2)
Toothache						1 (1)			
Puncture site inflammation									1 (1)
Conjunctivitis						1 (1)			
Blood uric acid increased						1 (1)			
C-reactive protein increased								3 (3)	
White blood cells urine-positive						1 (1)			
Headache					1 (1)	1 (1)			
Hypoesthesia	1 (1)								
Dermatitis									
Eczema nummular									1 (1)^*d*^
Nail discoloration								3 (3)	1 (1)^*d*^
Phlebitis								1 (1)	1 (1)

^*a*^ 1,800 mg twice daily on the first day, followed by 800 mg twice daily for the next 10 days.

^*b*^ 60-min intravenous administration throughout the treatment period.

^*c*^ During the treatment period, intravenous administration for the first 5 days, followed by a switch to oral administration.

^*d*^ Moderate TEAEs.

#### Single dose

No TEAEs were observed at ≤1,200 mg favipiravir. The incidence of TEAEs was 12.5% (one event) at 1,800 mg, 25.0% (five events) at 2,400 mg, and 10.0% (one event) in the placebo group. Of these, TEAEs related to favipiravir included one case of increased blood uric acid levels and one case of positive urinary leukocytes at 2,400 mg, although both were mild.

#### Multiple doses

The incidence of TEAEs for favipiravir was 55.6% (seven events) in the IV cohort, 77.8% (10 events) in the Switching cohort, and 33.3% (three events) in the placebo group. Of these, TEAEs judged related to favipiravir were three cases of nail discoloration in the IV cohort, four cases of nail discoloration, and one case of visual impairment in the Switching cohort. Visual impairment resolved on the day of onset. Nail discoloration was the most common TEAE observed in the multiple-dose trial. There was a pale yellowish color on the lunula of the nails, which resolved or mostly resolved 28–34 days after onset. All nail discolorations were mild.

Blood uric acid levels increased from Day 2 in favipiravir-treated subjects in both cohorts, reaching 11.3 mg/dL on Day 8 in the IV cohort and 12.5 mg/dL on Day 11 in the Switching cohort. Blood uric acid levels returned to normal promptly following completion of favipiravir administration. No TEAEs were suspected to be related to the elevated blood uric acid levels.

## DISCUSSION

The objective of this Phase I program was to characterize the pharmacokinetics and tolerability of intravenous favipiravir and to compare exposure with oral administration in healthy East Asian adults. This comparison should be interpreted with caution because favipiravir pharmacokinetics are nonlinear and may be influenced by the MBI of AO, body weight, acute illness and its severity, concomitant medications, and ethnicity.

In the power model analysis, neither the C_max_ nor the AUC_inf_ of favipiravir for intravenous administration showed dose linearity over the 300–2,400 mg dose range. In contrast, our in-house data showed that C_max_ and AUC_inf_ were linear over the dose range of 30–2,400 mg and 30–400 mg, respectively, when favipiravir was administered orally to healthy East Asian subjects. To clarify the dose ranges exhibiting linearity for intravenous administration, we conducted a *post hoc* analysis. As a result, while C_max_ exhibited linearity within the 300–1,200 mg dose range, AUC_inf_ did not demonstrate linearity across any dose range (Table 5). These findings suggest that concentration-dependent MBI of AO, a characteristic of favipiravir pharmacokinetics, may have exerted a stronger effect with intravenous administration.

**TABLE 5:** Assessment of dose-linearity on pharmacokinetic parameters of intravenous administration of favipiravir on each lower dose range

Dose range (mg)	Parameter		Slope (β_1_)	
		Estimate^*a*^	Standard error	95% confidence interval
300–2,400	C_max_	1.084	0.023	1.038–1.131
	AUC_inf_	1.822	0.085	1.649–1.995
300–1,800	C_max_	1.086	0.030	1.025–1.147
	AUC_inf_	1.700	0.085	1.526–1.874
300–1,200	C_max_	1.031	0.039	0.951–1.112
	AUC_inf_	1.448	0.097	1.247–1.650

^*a*^ Estimated regression slope using the power model.

**Fig 4:**
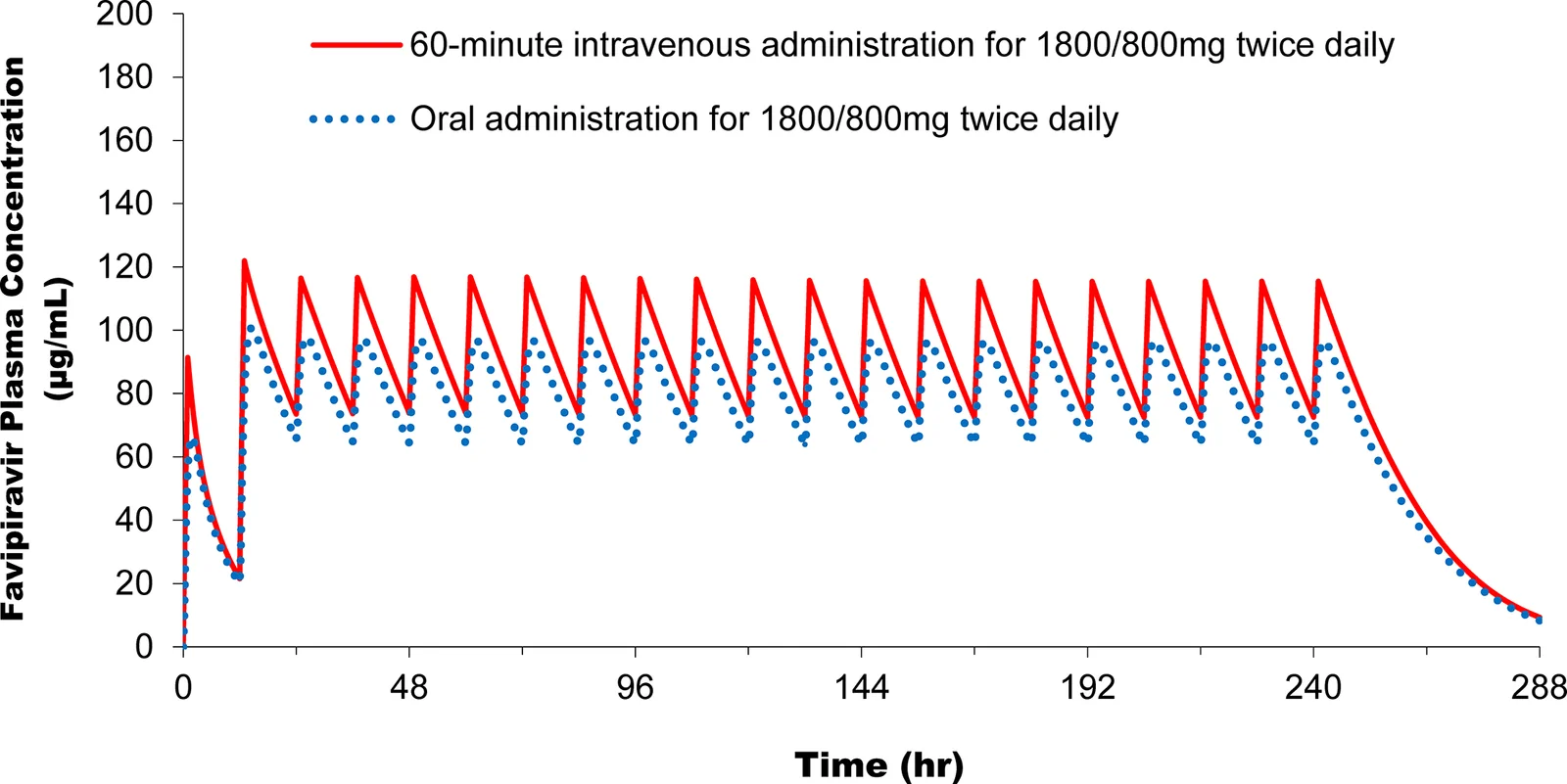
Expected plasma concentration-versus-time profile for favipiravir following intravenous and oral administration. Using the MBI-PK model, the predicted plasma concentration-versus-time profiles of favipiravir following 60-min intravenous and oral administration in subjects with standardized body weight are shown. Patients were administered 1,800 mg of favipiravir twice daily on the first day, followed by 800 mg twice daily for the subsequent 10 days. The dosing interval was 12 h. The red line represents plasma concentrations after multiple 60-min intravenous administrations, whereas the blue dashed line represents plasma concentrations after multiple oral administrations.

In the IV cohort of the multiple-dose trial, the geometric mean C_max_ and trough concentration (C_min_) were approximately 140 μg/mL and 85 μg/mL, respectively, on Days 6 and 11. The IV and Switching cohorts showed similar pharmacokinetic parameters during the treatment period. Compared with our in-house data from healthy East Asian subjects who received oral favipiravir 1,800/800 mg twice daily for 21 days, for which C_max_ and C_min_ after Day 2 were approximately 100 μg/mL and 60 μg/mL, respectively, intravenous administration produced exposures approximately 1.4-fold higher. Because favipiravir plasma concentrations are partly influenced by body weight (19), plasma concentration simulations using the MBI-PK model and standardized to the mean body weight of subjects in both administration-route groups were conducted. Although this adjustment diminished the difference between administration routes, plasma concentrations following intravenous administration were still estimated to be approximately 20% higher (Fig. 4).

Prior clinical experience with intravenous favipiravir remains limited. In a dose-escalating randomized Phase Ib study in hospitalized patients with COVID-19, intravenous favipiravir up to 2,400 mg twice daily was reported to be well tolerated, with no dose-limiting toxicities, expected transient hyperuricemia, disproportionate increases in exposure, and marked inter-individual pharmacokinetic variability (20). The exposures reported in that hospitalized COVID-19 population were not directly comparable with those in the present healthy-volunteer study. Differences in age, body weight, acute illness and disease severity, comorbidities, concomitant medications including drugs that may interact with favipiravir, and medical interventions could all contribute to the lower and more variable concentrations observed in patients (21). An ethnic component in favipiravir disposition or in the extent and recovery kinetics of AO cannot be excluded. This is because predominantly Caucasian patients with ambulatory influenza who received the same oral dosage as in the present multiple-dose trial had favipiravir concentrations less than half those observed here, whereas metabolite M1 concentrations remained comparable at approximately 3–4 μg/mL (19). However, ethnic differences in AO activity or in favipiravir-related MBI have not been established, and this hypothesis requires further evaluation. These pharmacokinetic comparisons should not be interpreted as evidence of clinical efficacy in COVID-19; controlled outpatient studies of oral favipiravir have not shown significant clinical or antiviral benefit (22).

During the maintenance dosing period in the present multiple-dose trial, the geometric mean C_min_ was approximately 80–85 μg/mL in both cohorts, and even the subject with the lowest concentration exceeded 35 μg/mL. Inter-individual variability remained large and was consistent with prior studies of favipiravir and M1 exposure (19, 20). This variability is likely related to substantial inter-individual differences in AO activity (23, 24) as well as body weight, disease status, and potential effects of concomitant medications and therefore cannot be fully resolved simply by using an injectable formulation. A prior study in ambulatory influenza reported that approximately 41%–43% of participants receiving the oral 1,800/800 mg twice-daily regimen had C_min_ values < 20 µg/mL during maintenance dosing, and reduced antiviral efficacy was observed in this subgroup (19). By contrast, the present intravenous regimen maintained concentrations above this exposure threshold in healthy East Asian subjects. Nevertheless, because the present study did not include patients with influenza or other acute viral infections, these PK/PD comparisons should be considered supportive for dose selection rather than proof of clinical efficacy.

Considering that the unbound fraction of favipiravir in human serum ranged from 0.456 to 0.466, a total trough concentration of approximately 85 μg/mL corresponds to an unbound concentration of approximately 39–40 μg/mL. *In vitro* susceptibility and *in vivo* antiviral effects differ substantially among RNA viruses; influenza viruses have generally shown greater susceptibility to favipiravir than coronaviruses, whereas other high-consequence RNA viruses require virus-specific interpretation. The unbound concentrations achieved in the present study were above reported IC_50_ values for seasonal influenza viruses, SFTS virus, Crimean-Congo hemorrhagic fever virus, and A/H5N1 influenza virus, but these comparisons do not directly establish clinical efficacy (7–10). Minimum effective doses of favipiravir in murine infection models for Crimean-Congo hemorrhagic fever virus and A/H5N1 influenza virus were lower than, or comparable to, those reported for the SFTS virus model that supported approval in Japan (8–10). These findings support further controlled studies rather than broad efficacy claims. Exploratory clinical trials of injectable favipiravir have been initiated in hospitalized older patients with seasonal influenza in Japan (jRCT identifier jRCT2031240586). In Crimean-Congo hemorrhagic fever, early-phase clinical evaluation has been conducted in UMIT-1 (ClinicalTrials.gov identifier NCT05940545), and a follow-up adaptive Phase IIb platform trial, UMIT-2, plans higher initial intravenous favipiravir doses of 2,600 mg twice daily on Day 1 and 1,200 mg twice daily on Day 2, followed by oral favipiravir 1,200 mg twice daily (ClinicalTrials.gov identifier NCT06860334).

Discoloration on the lunula of the nails in the multiple-dose trial was first noted after completion of dosing and occurred exclusively post-treatment. This phenomenon has already been reported as a TEAE associated with oral administration of favipiravir (25). Consistent with these findings, nonclinical studies have demonstrated that favipiravir administration induces discoloration of the fur and nails in animal models. Based on previous investigations using radioactive isotopes, the discoloration observed in the fur and nails of animals is thought to result from favipiravir-related substances that may bind to keratin or similar structural proteins. This discoloration was reversible after drug withdrawal and was not associated with histopathological abnormalities. The absence of this discoloration in the single-ascending-dose trial suggests that the event is exposure-dependent. Given this hypothesis, the higher incidence observed in the multiple-dose trial, which produced somewhat higher plasma concentrations than oral dosing, may also be explained. The visual impairment occurred approximately 2 h after the morning dose on Day 5 in the multiple-dose trial (Switching cohort). The investigator initially suspected homonymous hemianopia and consulted a neurosurgeon; however, neurological examination and brain magnetic resonance imaging revealed no abnormalities. Ophthalmological evaluation similarly showed no abnormalities. Both the neurosurgeon and the ophthalmologist considered migraine aura as a possible diagnosis, although the absence of headache symptoms made this uncertain. Transient occipital lobe ischemia was also considered, but coagulation parameters remained within normal ranges at all time points, including on the day of the event. This event resolved within a few hours. Further investigation involving additional cases is warranted to better characterize this event.

Our study has several limitations. First, the subjects were healthy, young East Asian adult males with relatively low body weight; therefore, the generalizability of the pharmacokinetic findings to older adults, females, non-Asian populations, patients with acute viral infections, and patients with higher body weight or severe disease is limited. Throughout the dosing period, the C_min_ of favipiravir administered orally at 1,800/800 mg twice daily to Japanese patients with SFTS was approximately 10 μg/mL lower than that observed in healthy subjects receiving the same oral dose, but no significant difference was noted. However, whether similar results would be obtained with intravenous administration is unclear. Second, the number of subjects receiving intravenous favipiravir was limited, with eight and nine subjects per dose regimen in the single-ascending dose and multiple-dose trials, respectively, resulting in a small sample size for evaluation. Because AO activity has substantial inter-individual variability (23), caution is warranted regarding the precision of the pharmacokinetic parameter estimates obtained in this study. Third, inter-individual variability remained large despite intravenous administration, and the present results do not eliminate the need for exposure monitoring or dose optimization in patient populations. Fourth, possible ethnic differences in favipiravir pharmacokinetics suggest that caution should be exercised when applying these parameters to non-Asian populations. Fifth, although no sex difference in AO activity has been reported (23), all subjects in this study were male, and no data are available for intravenous administration in East Asian females.

In summary, in healthy East Asian adult males, a single intravenous administration of favipiravir at 300–2,400 mg resulted in plasma concentrations comparable to those observed after a single oral administration. C_max_ and AUC_inf_ tended to increase with dose, although dose linearity could not be confirmed. After multiple intravenous administrations of favipiravir, plasma concentrations were approximately 20% higher than those achieved with the same oral doses after adjustment for body weight. Favipiravir administered at 1,800/800 mg twice daily for 11 days, either solely intravenously or by switching from intravenous to oral administration, maintained trough concentrations above the exposure threshold associated with antiviral activity in prior seasonal influenza PK/PD analyses. However, the clinical efficacy of intravenous favipiravir has not yet been established in controlled trials for influenza, Crimean-Congo hemorrhagic fever, or other serious RNA virus infections. The injectable formulation of favipiravir has the potential to become a valuable therapeutic option for patients with these RNA virus infections who have difficulty with oral administration. The results obtained from the present study support proceeding to clinical trials involving these patient populations.

## Data Availability

The data sets generated during and/or analyzed during the current study are still under the clinical development phase and are not publicly available. The data sets will be available from the sponsor on reasonable request after regulatory approval.

## SUPPLEMENTAL MATERIAL

Supplemental material aac.00463-26-s0001.docx Fig. S1 to S3; Tables S1 to S3.

ASM does not own the copyrights to Supplemental Material that may be linked to, or accessed through, an article. The authors have granted ASM a non-exclusive, world-wide license to publish the Supplemental Material files. Please contact the corresponding author directly for reuse.
