# Supplementary material for: Pharmacokinetics of injectable favipiravir: phase I, randomized, double-blind, placebo-controlled study in healthy East Asian subjects
Source: Antimicrob Agents Chemother. 2026 Jul 21;70(9):e00463-26. doi: 10.1128/aac.00463-26 (PMC13712965; doi:10.1128/aac.00463-26)
Supplement: Supplementary file 1 [file aac.00463-26-s0001.docx]

**Supplementary information**

**Fig. S1. Algorithm for subsequent dose determination in the single-ascending dose trial**

300 mg

60 min iv

300 mg

120 min iv

600 mg

120 min iv

1-a

Termination

Termination

1-c

600 mg

60 min iv

1-d

600 mg

120 min iv

900 mg

120 min iv

900 mg

60 min iv

1200 mg

60 min iv

1200 mg

120 min iv

1200 mg

120 min iv

1800 mg

60 min iv

2400 mg

60 min iv

2400 mg

120 min iv

Termination

Termination

Termination

Termination

2-a

2-b

2-c

2-d

3-a

3-c

4-a

4-b

Cohort-1

Cohort-2

Cohort-3

Cohort-4

Cohort-5

450 mg

120 min iv

Termination

1-b

1800 mg

120 min iv

Termination

3-b

iv, intravenous administration

(1) Criteria for selection of the dosage regimen in Cohort 2

iv, intravenous administration

1-a; If the mean C_max_ was ≥120 μg/mL

1-b; If the mean C_max_ was 100–<120 μg/mL

1-c; If the mean C_max_ was 80–<100 μg/mL

1-d; If the mean C_max_ was <80 μg/mL

(2) Criteria for selection of the dosage regimen in Cohort 3

iv, intravenous administration

2-a; If the C_max_ was ≥120 μg/mL

2-b; If the C_max_ was 100–<120 μg/mL

2-c; If the C_max_ was 80–<100 μg/mL

2-d; If the C_max_ was <80 μg/mL

(3) Criteria for selection of the dosage regimen in Cohort 4

3-a; If the C_max_ was ≥120 μg/mL

3-b; If the C_max_ was 100–<120 μg/mL

3-c; If the C_max_ was <100 μg/mL

(4) Criteria for selection of the dosage regimen in Cohort 5

4-a; If the C_max_ was ≥120 μg/mL

4-b; If the C_max_ was <120 μg/mL

**Table S1:**

**Observation and Examination Schedule in the single-ascending dose trial**

**(60-min infusion)**

| Time  (Day, hr)  Items | Day 1 | | | | | | | | | | Day 2 | | Day 3 | |
| --- | --- | --- | --- | --- | --- | --- | --- | --- | --- | --- | --- | --- | --- | --- |
|  | Predose | 0^a^ | 0.5 | 1 | 1.5 | 2 | 3 | 4 | 6 | 12 | 24 | 36 | 48 | 57 |
| Drug administration |  |  |  |  |  |  |  |  |  |  |  |  |  |  |
| Blood draw | X |  | X | X | X | X |  | X | X | X | X | X | X | X |
| Urine collection | X |  |  |  |  |  |  |  |  |  |  |  |  |  |
| Clinical findings | X |  |  | X |  | X |  | X |  | X | X | X | X | X |
| Vital signs | X |  |  | X |  | X | X | X | X | X | X | X | X |  |
| Electrocardiogram | X |  |  | X |  | X | X |  | X | X | X |  | X |  |
| Laboratory tests |  |  |  |  |  |  |  |  |  |  | X |  | X |  |

^a^ Time of dosing initiation was defined as 0 h.

**Table S2:**

**Observation and Examination Schedule in the single-ascending dose trial**

**(120-min infusion)**

| Time  (Day, hr)  Items | Day 1 | | | | | | | | | | Day 2 | | Day 3 | |
| --- | --- | --- | --- | --- | --- | --- | --- | --- | --- | --- | --- | --- | --- | --- |
|  | Predose | 0^a^ | 1 | 2 | 2.5 | 3 | 4 | 5 | 7 | 12 | 24 | 36 | 48 | 57 |
| Drug administration |  |  |  |  |  |  |  |  |  |  |  |  |  |  |
| Blood draw | X |  | X | X | X | X |  | X | X | X | X | X | X | X |
| Urine collection | X |  |  |  |  |  |  |  |  |  |  |  |  |  |
| Clinical findings | X |  |  | X |  | X |  | X |  | X | X | X | X | X |
| Vital signs | X |  |  | X |  | X | X | X | X | X | X | X | X |  |
| Electrocardiogram | X |  |  | X |  | X | X |  | X | X | X |  | X |  |
| Laboratory tests |  |  |  |  |  |  |  |  |  |  | X |  | X |  |

^a^ Time of dosing initiation was defined as 0 h.

**Table S3:**

**Observation and Examination Schedule in the multiple dose trial**

| Time  (Day, hr)  items | Day 1 | | | | | | | | | |
| --- | --- | --- | --- | --- | --- | --- | --- | --- | --- | --- |
|  | Predose | 0^a^ | 0.5 | 1 | 1.5 | 2 | 4 | 6 | 12 | 13 |
| Drug administration (IV) |  |  |  |  |  |  |  |  |  |  |
| Blood draw | X |  | X | X | X | X | X | X | X^b^ | X |
| Urine collection | X |  |  |  |  |  |  |  |  |  |
| Clinical findings | X |  |  | X |  | X | X |  | X^b^ | X |
| Vital signs | X |  |  | X |  | X | X |  | X^b^ | X |
| Electrocardiogram | X |  |  | X |  |  |  |  |  | X |

^a^ Initiation of the first investigational drug administration was designated as 0 h.

^b^ Assessment was conducted before the start of the second investigational drug administration.

| Time  (Day, hr)  Items | Days 2–5 | | | | | | | | | |
| --- | --- | --- | --- | --- | --- | --- | --- | --- | --- | --- |
|  | Predose | 0 ^a^ | 0.5 | 1 | 1.5 | 2 | 4 | 6 | 12 | 13 |
| Drug administration (IV) |  |  |  |  |  |  |  |  |  |  |
| Blood draw | X |  |  | X^b^ |  |  |  |  |  |  |
| Urine collection |  |  |  |  |  |  |  |  |  |  |
| Clinical findings | X |  |  | X |  |  |  |  |  | X |
| Vital signs | X |  |  | X |  |  |  |  |  |  |
| Electrocardiogram ^b^ | X |  |  | X |  |  |  |  |  |  |
| Laboratory tests ^b^ | X |  |  |  |  |  |  |  |  |  |

^a^ Initiation of the first investigational drug administration was designated as 0 h.

^b^ Days 2 and 5 only.

| Time  (Day, hr)  Items | Day 6 | | | | | | | | | |
| --- | --- | --- | --- | --- | --- | --- | --- | --- | --- | --- |
|  | Predose | 0 ^a^ | 0.5 | 1 | 1.5 | 2 | 4 | 6 | 12 | 13 |
| Drug administration (IV) ^b^ |  |  |  |  |  |  |  |  |  |  |
| Drug administration (Oral) ^c^ |  | X |  |  |  |  |  |  | X |  |
| Blood draw | X |  | X | X | X | X | X | X | X^d^ |  |
| Urine collection |  |  |  |  |  |  |  |  |  |  |
| Clinical findings | X |  |  | X |  | X | X |  | X^d^ | X |
| Vital signs | X |  |  | X |  | X | X |  | X^d^ | X |
| Electrocardiogram | X |  |  | X |  |  |  |  |  | X |

^a^ Initiation of the first investigational drug administration was designated as 0 h.

^b^ IV cohort.

^c^ Switching cohort.

^d^ Assessment was conducted before the start of the second investigational drug administration.

| Time  (Day, hr)  Items | Days 7–10 | | | | | | | | | |
| --- | --- | --- | --- | --- | --- | --- | --- | --- | --- | --- |
|  | Predose | 0 ^a^ | 0.5 | 1 | 1.5 | 2 | 4 | 6 | 12 | 13 |
| Drug administration (IV) ^b^ |  |  |  |  |  |  |  |  |  |  |
| Drug administration (Oral) ^c^ |  | X |  |  |  |  |  |  | X |  |
| Blood draw | X |  |  | X^d^ |  |  |  |  |  |  |
| Urine collection |  |  |  |  |  |  |  |  |  |  |
| Clinical findings | X |  |  | X |  |  |  |  |  | X |
| Vital signs | X |  |  | X |  |  |  |  |  |  |
| Electrocardiogram ^d^ | X |  |  | X |  |  |  |  |  |  |
| Laboratory tests ^d^ | X |  |  |  |  |  |  |  |  |  |

^a^ Initiation of the first investigational drug administration was designated as 0 h.

^b^ IV cohort.

^c^ Switching cohort.

^d^ Day 8 only

| Time  (Day, hr)  Items | Day 11 | | | | | | | | | 12 | 13 | 14 | Follow-up ^b^ | Discoloration  follow-up ^c^ |
| --- | --- | --- | --- | --- | --- | --- | --- | --- | --- | --- | --- | --- | --- | --- |
|  | Predose | 0 ^a^ | 0.5 | 1 | 1.5 | 2 | 4 | 6 | 12 | 24 | 48 | 72 |  |  |
| Drug administration (IV) ^b^ |  |  |  |  |  |  |  |  |  |  |  |  |  |  |
| Drug administration (Oral) ^c^ |  | X |  |  |  |  |  |  |  |  |  |  |  |  |
| Blood draw | X |  | X | X | X | X | X | X | X | X | X | X | X |  |
| Urine collection |  |  |  |  |  |  |  |  |  |  |  |  |  |  |
| Clinical findings | X |  |  | X |  |  |  |  |  | X | X | X | X |  |
| Discoloration findings ^d^ |  |  |  |  |  |  |  |  |  | X |  |  | X | X |
| Vital signs | X |  |  | X |  |  |  |  |  | X | X | X | X |  |
| Electrocardiogram | X |  |  | X |  |  |  |  |  |  |  |  | X |  |
| Laboratory tests | X |  |  |  |  |  |  |  |  |  |  | X | X |  |

^a^ Initiation of the first investigational drug administration was designated as 0 h.

^b^ Assessment was conducted on Days 19–21 or upon study discontinuation.

^c^ In the Switching cohort, the assessment was conducted 30 (±3) days after completion or discontinuation of the investigational drug administration.

^d^ Switching cohort only.

**Fig. S2. Structure of the physiologically based pharmacokinetic model of mechanical-based inhibition of aldehyde oxidase (MBI-PK model)**

C_p_=X2/V

CL=V_max_/(K_m_+C_p_)

dX1/dt= −Ka*X1

dX2/dt=K_a_*X1 − f_ao_*CL*C_p_*(E_act_/E_0_) − (1−f_ao_)* CL*C_p_

dE_act_/dt=K_deg_*(E_0_−E_act_) − (K_inact_*E_act_*f_p_*C_p_*K_p_)/(K’_app_+f_p_*C_p_*K_p_)

K_a_: First-order absorption rate constant

V_max_: Maximum metabolic velocity of favipiravir

K_m_: Michaelis–Menten constant

K_deg_: Rate constant for aldehyde oxidase (AO) enzyme synthesis

K_inact_: Maximum inactivation rate constant

K’_app_: Apparent dissociation constant between AO enzyme and favipiravir

E_act_: Active form of AO

E_0_: Total AO activity (fixed 1.0)

K_p_: Liver to plasma partition coefficients (fixed 0.266)

f_ao_: Contribution of AO to total clearance (fixed 0.841)

t_lag_: Lag-time (fixed 0.18)

E_0_ represents the total amount of activated AO at time zero, and E_act_ denotes the ratio of AO activated by favipiravir to E_0_. Therefore, E_0_ was fixed at 1.0, and K_p_ was set at 0.17 based on in-house data on tissue distribution and metabolism in rats following a single oral administration of ^14^C-favipiravir.

f_ao_ was calculated from the difference in the clearance of M1 between the first and last administrations of favipiravir in healthy subjects under the following assumptions: AO was completely inactivated at the last administration, favipiravir was completely absorbed, M1 clearance via AO was equal to favipiravir oral clearance, and the clearance of M1 was unchanged by multiple administrations.

f_p_ included in E_act_ was set at 0.462 based on in-house data from a serum protein-binding study. The t_lag_ was estimated at 0.18 by one-compartmental analysis using data from multiple oral administration studies of favipiravir (in-house data).

**Fig. S3. Assessment of MBI-PK model**

The relationship between observed concentrations for each subject in the multiple dose trial and the corresponding predicted values estimated using the MBI-PK model. For both the IV (orange) and Switching (blue) cohorts, the observed and predicted values were generally consistent.
